# bayes4psy—An Open Source R Package for Bayesian Statistics in Psychology

**DOI:** 10.3389/fpsyg.2020.00947

**Published:** 2020-05-12

**Authors:** Jure Demšar, Grega Repovš, Erik Štrumbelj

**Affiliations:** ^1^Faculty of Computer and Information Science, University of Ljubljana, Ljubljana, Slovenia; ^2^Mind & Brain Lab, Department of Psychology, Faculty of Arts, University of Ljubljana, Ljubljana, Slovenia

**Keywords:** Bayesian statistics, R, psychology, reaction time, success rate, Bayesian *t*-test, color analysis, linear model

## Abstract

Research in psychology generates complex data and often requires unique statistical analyses. These tasks are often very specific, so appropriate statistical models and methods cannot be found in accessible Bayesian tools. As a result, the use of Bayesian methods is limited to researchers and students that have the technical and statistical fundamentals that are required for probabilistic programming. Such knowledge is not part of the typical psychology curriculum and is a difficult obstacle for psychology students and researchers to overcome. The goal of the bayes4psy package is to bridge this gap and offer a collection of models and methods to be used for analysing data that arises from psychological experiments and as a teaching tool for Bayesian statistics in psychology. The package contains the Bayesian *t*-test and bootstrapping along with models for analysing reaction times, success rates, and tasks utilizing colors as a response. It also provides the diagnostic, analytic and visualization tools for the modern Bayesian data analysis workflow.

## 1. Introduction

Bayesian data analysis with custom models offers a highly flexible, intuitive and transparent alternative to classical statistics. Throughout much of the modern era of science Bayesian approaches were on the sidelines of data analysis, mainly due to the fact that computations required for Bayesian analysis are usually quite complex. But computations that were only a decade or two ago too complex for specialized computers can now be executed on average desktop computers. In part also due to modern Markov chain Monte Carlo (MCMC) methods that make computations tractable for most parametric models. This, along with specialized probabilistic programming languages for Bayesian modeling, such as Stan (Carpenter et al., 2017) and JAGS (Plummer, 2003), drastically increased the accessibility and usability of Bayesian methodology for data analysis. Indeed, Bayesian data analysis is steadily gaining momentum in the twenty-first century (Gelman et al., 2014; Kruschke, 2014; McElreath, 2018), especially so in natural and technical sciences. Unfortunately, the use of Bayesian data analysis in social sciences remains scarce, most likely due to a steep learning curve associated with Bayesian analysis.

There are many advantages of Bayesian data analysis (Dunson, 2001; Gelman et al., 2014; Kruschke, 2014; McElreath, 2018), such as its ability to work with missing data and incorporating prior information about the data in a natural and principled way. Furthermore, Bayesian methods offer high flexibility through hierarchical modeling, while calculated posterior parameter values can be used as easily understandable alternatives to *p*-values. Bayesian methods provide very intuitive and interpretable answers, such as “the parameter μ has a probability of 0.95 of falling inside the [−2, 2] interval.”

One of the social sciences that can substantially benefit from Bayesian methodology is psychology. The majority of data that are acquired in psychological experiments, such as reaction times, success rates, and picked colors, can be analyzed in a Bayesian manner by using a small set of probabilistic models. To a certain degree Bayesian methodology could also alleviate the replication crisis that is pestering the field of psychology (Schooler, 2014; Open Science Collaboration, 2015; Stanley et al., 2018).

The ability to replicate scientific findings is of paramount importance to scientific progress (McNutt, 2014; Baker and Penny, 2016; Munafò et al., 2017). Unfortunately, more and more replications fail to reproduce original results and conclusions (Schooler, 2014; Open Science Collaboration, 2015; Amrhein et al., 2019). This so-called replication crisis is not only harmful to the authors of such studies but to science itself. A recent attempt to replicate 100 studies from three prominent psychology journals (Open Science Collaboration, 2015) showed that only approximately a third of studies that claimed statistical significance (*p*-value < 0.05) also showed statistical significance in replication. Another recent study (Camerer et al., 2018) tried to replicate systematically selected studies in the social sciences published in Nature and Science between 2010 and 2015, replication attempts were successful only in 13 out of 21 cases.

The main reasons behind the replication crisis seem to be poor quality control in journals, unclear writing and inadequate statistical analysis (Wasserstein and Lazar, 2016; Hurlbert et al., 2019; Wasserstein et al., 2019). One of the fundamental issues lies in the desire to claim statistical significance through *p*-values. Many manuscripts published today repeat the same mistakes even though prominent statisticians prepared extensive guidelines on what to do and mainly what not to do (Hubbard, 2015; Wasserstein and Lazar, 2016; Wasserstein et al., 2019; Ziliak, 2019). Reluctance to adhere to modern statistical practices has led scientist to believe that a more drastic shift in statistical thinking is needed, and some believe that it might come in the form of Bayesian statistics (Dunson, 2001; Gelman et al., 2014; Kruschke, 2014; McElreath, 2018).

Some software tools and packages already bring Bayesian statistics to broader audiences. JASP (Love et al., 2019) is a graphical statistical software that also implements Bayesian alternatives for some common statistical tests (e.g., *t*-test, ANOVA, …). JASP allows execution of statistical analyses through its highly intuitive graphical user interface. Another great tool for executing elementary Bayesian analyses is Rasmus Bååth's BayesianFirstAid (Bååth, 2014). The goal of this R package is to replace the classic elementary statistical tests with their Bayesian counterparts. Since both JASP (Love et al., 2019) and BayesianFirstAid (Bååth, 2014) focus on the most elementary statistical tests, the tools they offer are often insufficient when working with more complex data sets. The development of a package that would cover all needs of modern science is impossible, but as a subset of specialized Bayesian models is sufficient to cover the majority of analyses in psychology, we developed the bayes4psyR package.

The bayes4psyR package provides a state-of-the art framework for Bayesian analysis of psychological data. It incorporates a set of probabilistic models for analysing data that arise during many types of psychological experiments. All models are pre-compiled, meaning that we do not need any specialized software or skills (e.g., knowledge of probabilistic programming languages). The only requirements are the R programming language and very basic programming skills (same skills as needed for classical statistical analysis in R). The package also incorporates the diagnostic, analytic and visualization tools required for modern Bayesian data analysis. The bayes4psy package represents a bridge into the exciting world of Bayesian statistics for students and researches in the field of psychology.

## 2. Methods and Models

For statistical computation (sampling from the posterior distributions) the bayes4psy package utilizes Stan (Carpenter et al., 2017). Stan is a state-of-the-art platform for statistical modeling and high-performance statistical computation and offers full Bayesian statistical inference with MCMC sampling. It also offers friendly interfaces with most programming languages used for statistical analysis, including R. R (R Core Team, 2017) is one of the most powerful and widespread programming languages for statistics and visualization. Visualizations in the bayes4psy package are based on the ggplot2 package (Wickham, 2009).

Bayesian analysis requires three key pieces of information—the input data, the statistical model and the priors. By far the most complex of the three is the development of a statistical model, which requires extensive knowledge in probabilistic programming. To avoid this difficult step, the bayes4psy package includes an already prepared collection of models for analysing the most common types of data arising from psychological research.

### 2.1. The Input Data

In psychology and many other scientific fields data are typically gathered with experiments, surveys, questionnaires, observations, and other similar data collection methods. Gathering and preparing the data for use with the bayes4psy package is the same as for any other statistical analysis.

### 2.2. The Statistical Model

The bayes4psy package contains a collection of Bayesian models suitable for analysing common types of data that arise during psychological experiments. The packages includes the Bayesian *t*-test and bootstrap and models for analysing reaction times, success rates, and tasks utilizing colors as a response. Besides the models, we also prepared the diagnostic, analytic, and visualization tools for the modern Bayesian data analysis workflow.

Statistical models are defined through distributions and their parameters. For example, the Bayesian *t*-test utilizes a generalized *t*-distribution which has three parameters—degrees of freedom ν, location/mean μ, and scale/variance σ. In the remainder of the paper, we describe and visualize all the models in the bayes4psy package.

### 2.3. Priors

In Bayesian statistics we use prior probability distributions (priors) to express our beliefs about the model's parameters before any evidence (data) is taken into account. Priors represent an elegant way of combining (pre)existing knowledge with new facts about the domain of analysis. Prior distributions are usually based on past research or domain expertise. If prior information is unavailable, we usually resort to weakly informative, vague priors. We can also leverage prior information to increase the power of small-sample studies.

In the bayes4psy package we can express prior knowledge with prior distributions on all of the model's parameters. The package supports uniform, normal, gamma and beta prior distributions. By default flat/improper priors are used for all of the model's parameters. For details, see the illustrative examples in section 3.

### 2.4. Outputs

The outputs of the MCMC-based Bayesian inference are samples. These samples represent credible values for parameters of the chosen statistical model. For example, the samples of the Bayesian *t*-test model contain values for the parameters of the underlying *t*-distribution—degrees of freedom ν, mean μ, and variance σ. Once we acquire these samples, typically hundreds or thousands of them, we can use them for statistical inference. The samples can be used in a number of ways, for example, we can use them to compare means of two or more groups, we can reconstruct the estimated distribution of the population, we can describe the group by calculating summary statistics (e.g., mean, confidence interval) of certain parameters.

### 2.5. A Simplified Example

Suppose we are interested in comparing the mean heights of Europe and US primary school pupils. First, we need to define our inputs—the input data, the statistical model and the priors. The input data are the actual height measurements of the pupils. Next, we have to pick an appropriate model. Since we are interested in comparison of the means, we can use the model for the Bayesian *t*-test (see the section 2.6 for a detailed explanation of this model). This model has three parameters—degrees of freedom ν, mean μ, and variance σ. We can specify priors for these parameters or use the default non-informative priors. An example of a weakly informative or vague prior in this example would be a uniform distribution U(0,200) for the μ parameter. With this prior on μ we are postulating that mean height of primary school pupils lies strictly somewhere between 0 and 200 cm. Priors can be based on previous studies or expert knowledge. For example, since mean height of primary school pupils is around 120 ± 20 cm a reasonable informative prior for the μ parameter could be N(120,20). In a similar way we can define priors for ν and σ.

Once we have selected the priors, we are ready to infer the distributions underlying the chosen model (fit the model) to our data for each of the two groups (height of pupils in Europe and height of pupils in USA). The output of the inference process are the generated samples of the model's parameters. Suppose that the generated samples are μ_*EU*_ = [123, 128, 121, 137, 110 cm] and μ_*US*_ = [118, 126, 119, 110, 122 cm]. We can compare the mean height of these two groups by executing a pair-wise comparison of the μ samples. In this example we can claim with 80% certainty that European pupils are higher than their US counterparts (in four out of five samples, the μ parameter of European pupils is higher—123 > 118 cm, 128 > 126, 121 > 119 cm, 137 > 110 cm, 110 < 122 cm). Note that in practice we would typically have hundreds or thousands of samples.

We can also check if means of two groups is equal. One way of doing this is by defining the ROPE (Region Of Practical Equivalence) interval. For example, if our measuring equipment had a tolerance of 0.2 cm, then it would make sense to set the ROPE interval to [−0.2, 0.2]. Samples from both groups that differ for <0.2 cm would be interpreted as equal and we would be able to compute the probability that the means are (practically) equal.

### 2.6. Bayesian *t*-Test

**Figure 1:**
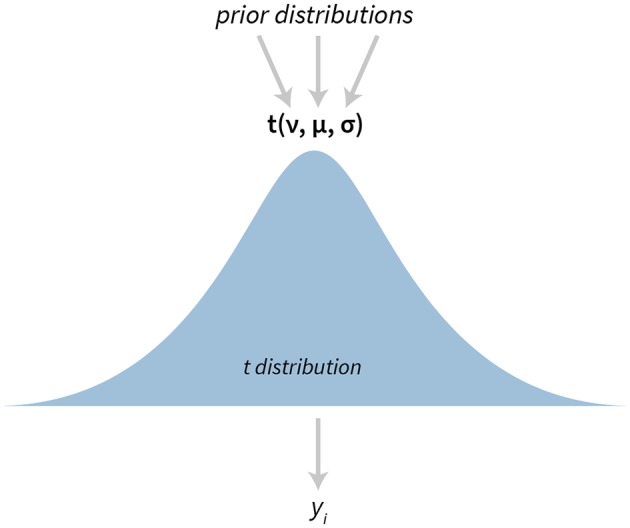
The visualization of the Bayesian *t*-test. The model has three parameters—degrees of freedom ν, mean μ, and variance σ. *y*_*i*_ denotes *i*-th datum in the provided data set.

The *t*-test is one of the most popular statistical tests. In bayes4psy it is based on Kruschke's model (Kruschke, 2013, 2014) which uses a scaled and shifted Student's *t*-distribution (Figure 1). This distribution has three parameters—degrees of freedom (ν), mean (μ), and variance (σ).

There are some minor differences between our implementation and Kruschke's. Instead of pre-defined vague priors for all parameters, we can define custom priors for the ν, μ, and σ. Kruschke's implementation models two data sets simultaneously, while in bayes4psy we can model several data sets individually and then make pairwise comparisons or a simultaneous cross comparison between multiple fits. We illustrate the use of the *t*-test in section 3.3.

### 2.7. Model for Analysing Reaction Times

Psychological experiments typically have a hierarchical structure—each subject (participant) performs the same test for a number of times, several subjects are then grouped together by their characteristics (e.g., by age, sex, health) and the final statistical analysis is conducted at the group level. Such structure is ideal for Bayesian hierarchical modeling (Kruschke, 2014).

Our subject-level reaction time model is based on the exponentially modified normal distribution. This distribution has proven to be a suitable interpretation for the long tailed data that arise from reaction time measurements Lindeløv (2019). Note here, that the exponentially modified normal distribution is flexible and can also accommodate the cases in which data are distributed normally. To model the data at the group level we put hierarchical normal priors on all parameters of the subject-level exponentially modified normal distribution.

**Figure 2:**
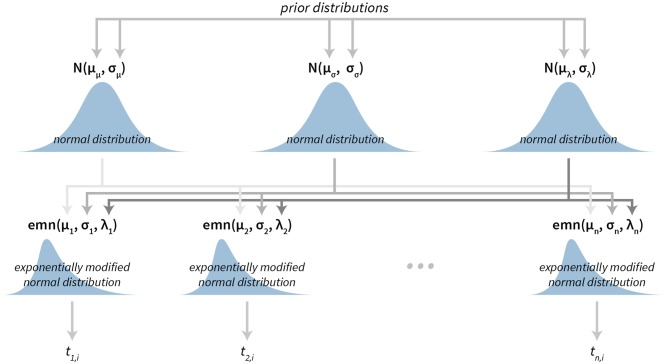
The visualization of the Bayesian reaction time model. The model has a hierarchical structure. Reaction times belonging to each individual subject (*t*_*n, i*_ depicts *i*-th reaction time of the subject *n*) are used to construct exponentially modified normal distributions at the subject level. Parameters of subject level distributions are then connected at the group level by using normal distributions, which can then be used for group level analysis.

The subject level parameters are thus μ_*i*_, σ_*i*_, and λ_*i*_, where *i* is the subject index. And hierarchical normal priors on these parameters are $N\left(\mu_{\mu },\sigma_{\mu }\right)$ for the μ parameter, $N\left(\mu_{\sigma },\sigma_{\sigma }\right)$ for the σ parameter and $N\left(\mu_{\lambda },\sigma_{\lambda }\right)$ for the λ parameter. See Figure 2 for a graphical representation of the Bayesian reaction time model. For a practical application of this model see section 3.1.

In the case of an exponentially modified normal distribution means are calculated using the μ and λ parameters. By default, bayes4psy reports means on the group level, calculated as *E* = μ_μ_+1/μ_λ_.

### 2.8. Model for Analyzing Success Rates

The success rate model is based on the Bernoulli-Beta model that can be found in most Bayesian statistics textbooks (Gelman et al., 2014; Kruschke, 2014; McElreath, 2018). This model is used for modeling binary data. In our case this binary output represents whether a subject successfully solved the given task or not.

**Figure 3:**
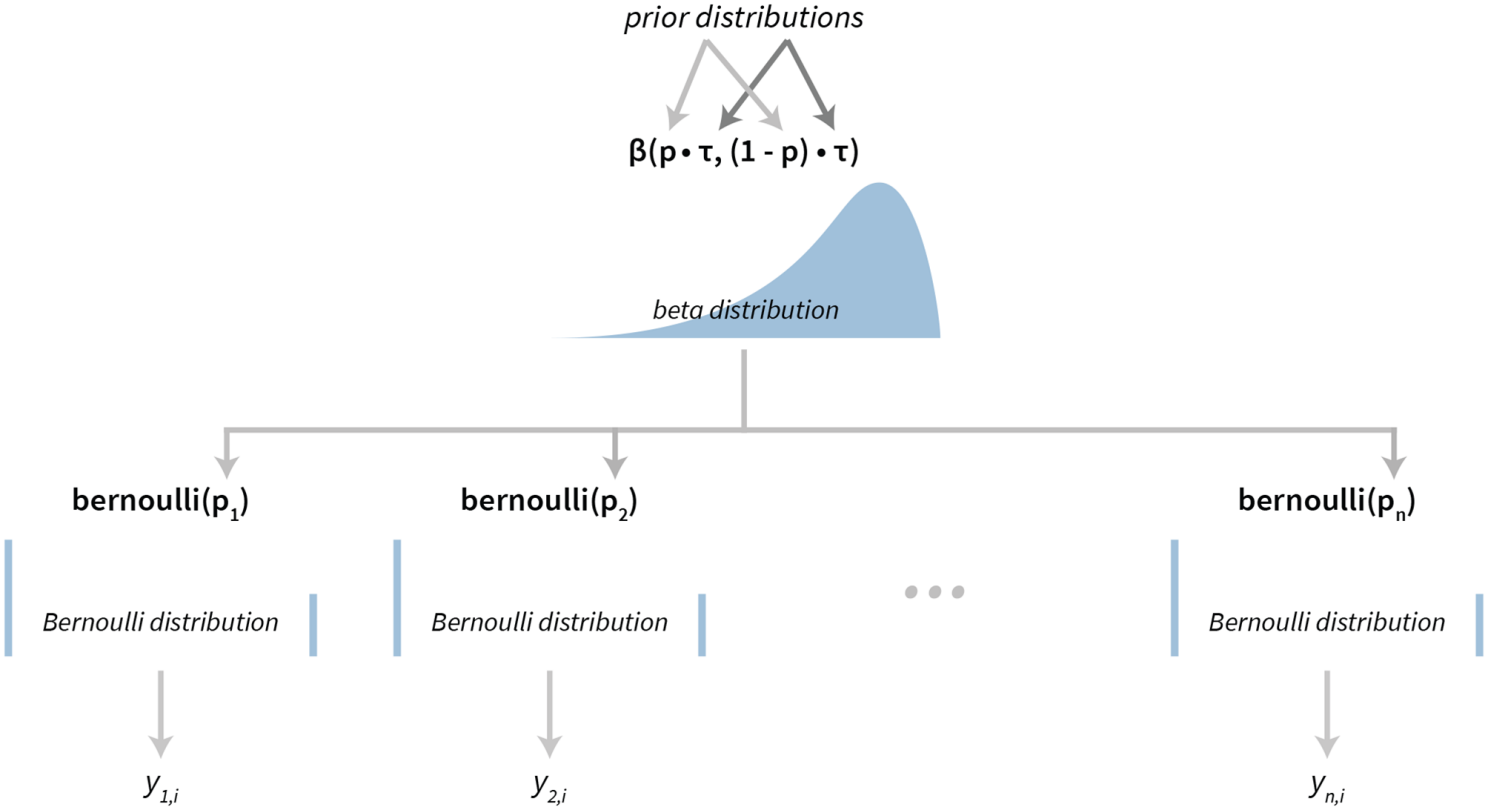
The visualization of the Bayesian success rate model. The model has a hierarchical structure. Data about success of individual subjects (*y*_*n, i*_ depicts success on the *i*-th attempt of the subject *n*) are used for inferring Bernoulli distributions on the subject level. Parameters of subject level distributions are then connected at the group level with a Beta distribution.

The success rates model also has a hierarchical structure. The success rate of individual subjects is modeled using Bernoulli distributions, where the *p*_*i*_ is the success rate of subject *i*. A reparameterized Beta distribution, Beta(*pτ*, (1−*p*)τ), is used as a hierarchical prior on subject-level parameters, where *p* is the group level success rate and τ is the scale parameter. A graphical representation of our hierarchical success rate model can be seen in Figure 3. For a practical application of this model see section 3.1.

### 2.9. Model for Analysis of Sequential Tasks

In some psychological experiments data have a time component or some other ordering. For example, when subjects are asked to perform a sequence of tasks. To model how a subject's performance changes over time, we implemented a hierarchical linear normal model.

The sequence for a subject is modeled using a simple linear model with subject-specific slope and intercept. To model the data at the group level we put hierarchical normal priors on all parameters of the subject-level linear models. The parameters of subject *i* are α_*i*_ for the intercept, β_*i*_ for the slope and σ_*i*_ for modeling errors of the fit (residuals). The hierarchical normal priors on these parameters are $N\left(\mu_{\alpha },\sigma_{\alpha }\right)$ for the intercept α, $N\left(\mu_{\beta },\sigma_{\beta }\right)$ for the slope β and $N\left(\mu_{\sigma },\sigma_{\sigma }\right)$ for the residuals (σ).

**Figure 4:**
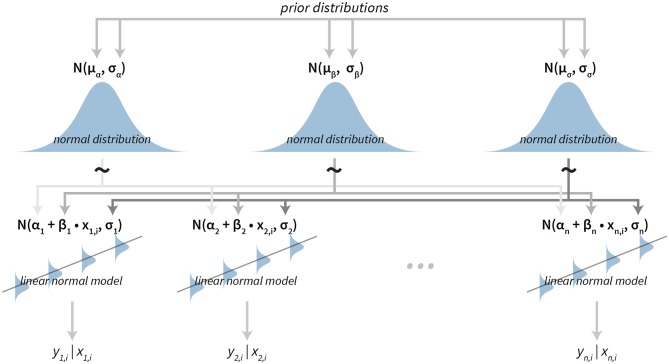
The visualization of the hierarchical linear model. The model has a hierarchical structure, linear normal models are fitted on the subject level from data belonging to each particular subject. Since the ordering is important input data come in pairs of dependent (e.g., result or answer) and independent variables (e.g., time or the question index). The term *y*_*n, i*_|*x*_*n, i*_ is the value of the *i*-th dependent variable given the value of the independent variable *i* for the subject *n*. Parameters of subject level distributions are joined on the group level by using normal distributions. These group level distributions can then be used for group level analysis of the data.

A graphical representation of the model is shown in Figure 4. For a practical application of this model see section 3.2.

### 2.10. Model for Analysis of Tasks Utilizing Colors as a Response

This model is designed for experiments in which subject's response comes in the form of a color (e.g., subjects have to pick a color that describes their mood, subject have to remember a color and then pick it from a color palette after a certain time interval …). Color stimuli and subject responses in psychological experiments are most commonly defined through the RGB color model. The name of the model comes from the initials of the three additive primary colors, red, green, and blue. These colors are also the three components of the model, where each component has a value ranging from 0 to 255 which defines the presence of a particular color. Since defining and analysing colors through the RGB model is not very user friendly and intuitive, our Bayesian model is capable of working with both the RGB and HSV color models. HSV (hue, saturation and value) is an alternative representation of the RGB model that is usually easier to read and interpret for most human beings.

**Figure 5:**
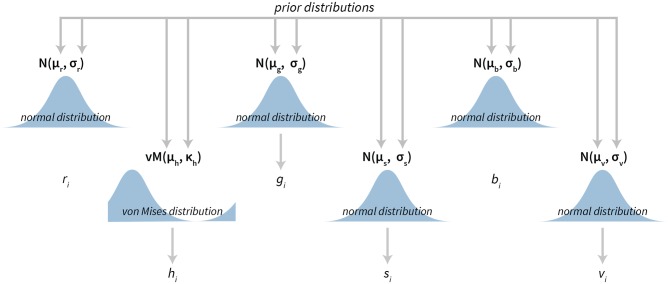
The visualization of the Bayesian color model. The model is composed of six parts. Three parts are used to describe the RGB (red, green, blue) color model components and three parts are used to describe the HSV (hue, saturation, value) color model components. All components, except hue, are modeled with normal distributions, while hue is modeled with the von Mises distribution—a circular normal distribution.

The Bayesian color model works in a component-wise fashion. Six distributions (three for the RGB components and three for the HSV components) are inferred from the data for each component individually. For RGB components we use normal distributions (truncated to the [0, 255] interval). In the HSV case, we used [0, 1]-truncated normal distributions for saturation and value components and the von Mises distribution for the hue component. The von Mises distribution (also known as the circular normal distribution) is a close approximation to the normal distribution wrapped on the [0, 2π] interval. A visualization of our Bayesian model for colors can be seen in Figure 5 and its practical application in section 3.4.

### 2.11. The Bayesian Bootstrap

The bootstrap is a resampling technique for computing standard deviations, confidence intervals and other estimates for quantifying uncertainty. It uses sampling with replacement to approximate the sampling distribution of an estimator and is applicable in a uniform way to a wide range of scenarios.

The Bayesian bootstrap in bayes4psy is the analog of the classical bootstrap (Efron, 1979). It is based on Rasmus Bååth's implementation (Bååth, 2015), which in turn is based on methods developed by Rubin (1981). The Bayesian bootstrap does not simulate the sampling distribution of a statistic estimating a parameter, but instead simulates the posterior distribution of the parameter. The statistical model underlying the Bayesian bootstrap can be characterized by drawing weights from a uniform Dirichlet distribution with the same dimension as the number of data points. These draws are then used for calculating the statistic in question and weighing the data (Bååth, 2015). For more details about the implementation see Bååth (2015) and Rubin (1981).

### 2.12. Methods for Fitting and Analysing Bayesian Fits

This section provides a quick overview of all the methods for fitting and analysing the models described in previous sections. For a more detailed description of each function we invite the reader to consult the bayes4psy package documentation and examples.

The first set of functions infers the parameters of model's distributions from the input data, in other words these functions fit the model to the data. We can also use these functions to define priors (for an example, see the second part of section 3.1) or configure the fitting parameters. This way we can set the number of generated samples (number of MCMC iterations) along with several other parameters of the MCMC algorithm. Some basic MCMC settings are described in this manuscript and the documentation of this package, for more advanced settings consult the official Stan documentation (Carpenter et al., 2017).

- b_ttest is used for fitting the Bayesian *t*-test model. The input data comes in the form of a vector of normally distributed real numbers.
- b_linear is used for fitting the hierarchical linear model, suitable for analysing sequential tasks. The input data are three vectors—*x* a vector containing values of the independent variable (time, question index …), *y* a vector containing values of the dependent variable (subject's responses) and *s* a vector containing IDs of subjects, these IDs are used for denoting that *x*_*i*_/*y*_*i*_ pair belongs to a particular subject.
- b_reaction_time is used for the Bayesian reaction time model. Its input data are two vectors—vector *t* includes reaction times while vector *s* is used for linking reaction times with subjects.
- b_success_rate is used for fitting the Bayesian success rate model. Its input data are two vectors, the first vector *r* contains results of an experiment with binary outcomes (e.g., success/fail, hit/miss …) and the second vector *s* is used to link these results to subjects.
- b_color is used for fitting the color model. The input data to this model is a three column matrix or a data.frame where each column represents one of the components of the chosen color model (RGB or HSV). If the input data are provided in the HSV format then we also have to set the *hsv* parameter to TRUE.
- b_bootstrap function can be used for Bayesian bootstraping. The input data can be in the form of a vector, matrix or a data.frame. The Bayesian bootstrap also requires the specification of the statistics function.

Before interpreting the results, we can use the following functions to check if the model fits are a credible representation of the input data:

- plot_trace draws the Markov chain trace plot for main parameters of the model, providing a visual way to inspect sampling behavior and assess mixing across chains and convergence.
- plot or plot_fit draws the inferred distributions against the input data. With hierarchical models we can use the subjects parameter to draw fits on the subject level.
- plot_hsv or plot_fit_hsv are special functions for inspecting color model fits by using a color wheel visualization of HSV components.

For a summary of the posterior with Monte Carlo standard errors and confidence intervals we can use the summary or print/show functions:

- summary prints summary statistics of the main model's parameters.
- print, show prints a more detailed summary of the model's parameters. It includes estimated means, Monte Carlo standard errors (se_mean), confidence intervals, effective sample size (n_eff, a crude measure of effective sample size), and the R-hat statistic for measuring auto-correlation. R-hat measures the potential scale reduction factor on split chains and equals 1 at convergence (Gelman and Rubin, 1992; Brooks and Gelman, 1998).

The compare_means function can be used for comparison of parameters that represent means of the fitted models. To visualize these means one can use the plot_means function and for visualizing the difference between means the plot_means_difference function. All comparison functions (functions that print or visualize the difference between fitted models) also offer the option of defining the ROPE interval by setting the rope parameter.

- compare_means prints and returns a data.frame containing the comparison. It can be used for comparing two or multiple models at the same time.
- plot_means_difference visualizes the difference of means between two or multiple models at the same time.
- plot_means plots the distribution of parameters that depict means. It can be used on a single or multiple models at the same time.
- plot_means_hsv is a special function for the Bayesian color model that plots means of HSV components by using a color wheel visualization.

The following set of functions works in a similar fashion as the one for comparing means, the difference is that this one compares entire distributions and not just the means. This analysis is based on the comparison of a large amount of samples drawn from the distributions.

- compare_distributions prints and returns a data.frame containing the comparison results. It can be used for comparing two or multiple models at the same time.
- plot_distributions_difference visualizes the difference of distributions underlying two or multiple fits at the same time.
- plot_distribution plots the distributions underlying the fitted models, can be used on a single or multiple models at the same time.
- plot_distributions_hsv is a special function for the Bayesian color model that plots the distribution behind HSV components by using a color wheel like visualization.

We can also extract samples from the posterior for further custom analyses:

- get_parameters returns a data.frame of model's parameters. In hierarchical models this returns a data.frame of group level parameters.
- get_subject_parameters can be used to extract subject level parameters from hierarchical models.

## 3. Illustrative Examples

For the sake of brevity, we are presenting diagnostic visualizations and outputs only the first time they appear and omit them in later examples. The datasets used in the examples are based on the experiments conducted by the Mind & Brain Lab at the Faculty of Arts, University of Ljubljana. All datasets are included in the bayes4psy package.

### 3.1. The Flanker Task

In the Eriksen flanker task (Eriksen and Eriksen, 1974) participants are shown an image of an odd number of arrows (usually five or seven). Their task is to indicate the orientation (left or right) of the middle arrow as quickly as possible. There are two types of stimuli: in the *congruent* condition (e.g., “<<<<<<<”) both the middle arrow and the flanking arrows point in the same direction and in the *incongruent* condition (e.g., “<<<><<<”) where the middle arrow points in the opposite direction.

The participants have to consciously ignore and inhibit the misleading information provided by the flanking arrows in the incongruent condition, which leads to robustly longer reaction times and a higher proportion of errors. The difference between reaction times and error rates in congruent and incongruent conditions is a measure of the subject's ability to focus and to inhibit distracting stimuli.

In the illustration below we compare reaction times and error rates when performing the flanker task between the control group (healthy subjects) and the test group (subjects suffering from a certain medical condition).

First, we load bayes4psy and dplyr (Wickham et al., 2018) for data wrangling. Second, we load the data and split them into control and test groups. For reaction time analysis we use only data where the response to the stimuli was correct:


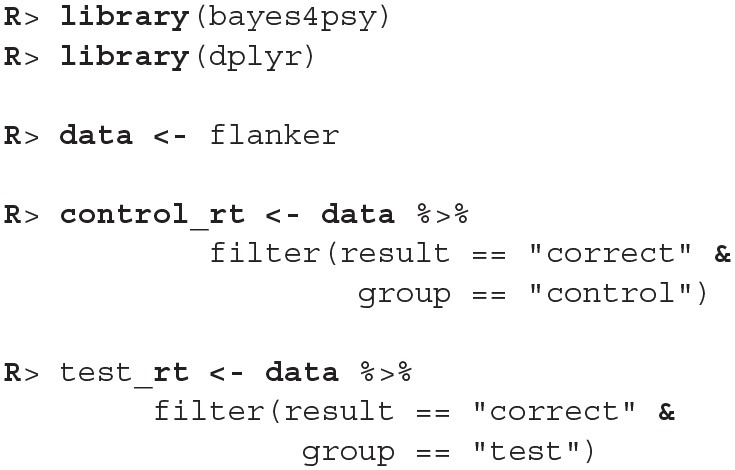


The model requires subjects to be indexed from 1 to *n*. Control group subject indexes range from 22 to 45, so we have to cast them to an interval that ranges from 1 to 23. Note here, that even though this way both control and test subject have some indexes, they will be still treated as separate individuals because the models for test and control subjects will be fitted separately.


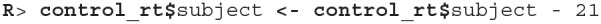


Now we are ready to fit the Bayesian reaction time model to data from both groups. The modeling function (b_reaction_time) requires two parameters—a vector of reaction times *t* and the vector of subject indexes *s*.


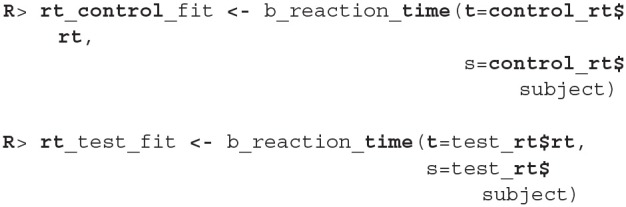


**Figure 6:**
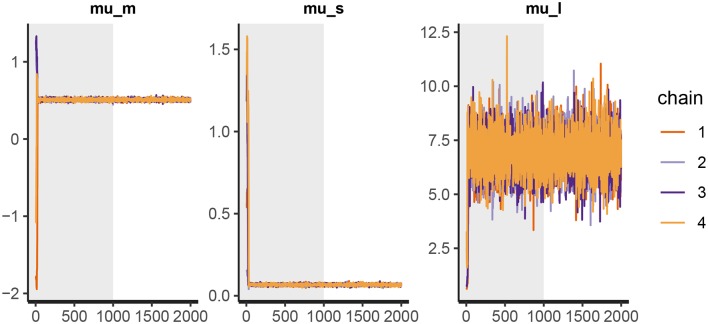
The trace plot for rt_control_fit. The traceplot gives us no cause for concern regarding MCMC convergence and mixing. The trace plot for rt_test_fit is similar. Note that the first 1,000 iterations (shaded gray) are used for warmup (tuning of the MCMC algorithm) and are discarded. The next 1,000 iterations are used for sampling. Informally speaking, if trace plots after the warmup period look like “hairy caterpillars” there is no reason for concern.

Before we interpret the results, we check MCMC diagnostics (such as the traceplot on Figure 6, the Rhat metric and the effective sample size) and inspect model's fit.


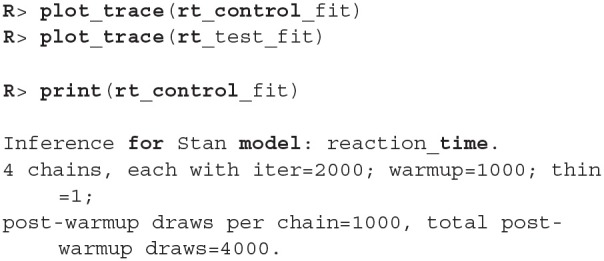



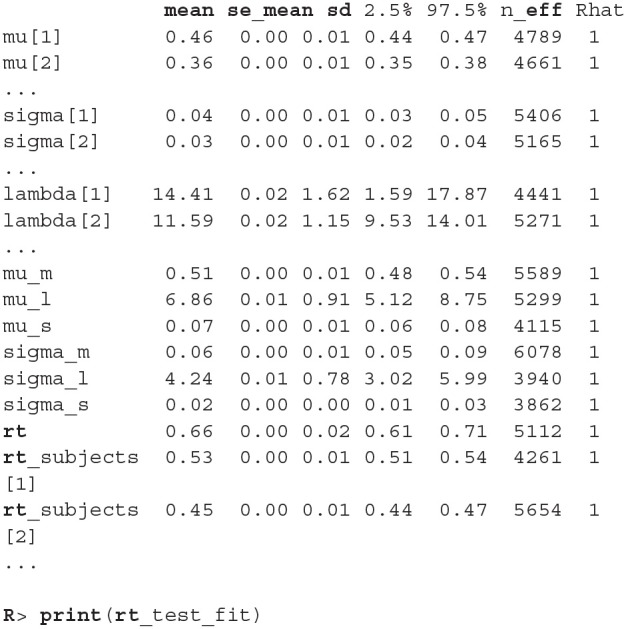


The output above is truncated and shows only values for 2 of the 24 subjects on the subject level of the hierarchical model. The output provides further MCMC diagnostics, which again do not give us any cause for concern. The convergence diagnostic Rhat is practically 1 for all parameters and there is little auto-correlation—effective sample sizes (n_eff) are of the order of samples taken and Monte Carlo standard errors (se_mean) are relatively small.

What is a good-enough effective sample sizes depends on our goal. If we are only interested in estimating the mean, 100 effective samples is in most cases enough for a practically negligible Monte Carlo error. On the other hand if we are interested in posterior quantities, such as extreme percentiles for example, the effective sample sizes might have to be 10,000 or higher.

We can increase the effective sample size by increasing the amount of MCMC iterations with the iter parameter. In our case we can achieve an effective sample size of 10,000 by setting iter to 4,000. Because the MCMC diagnostics give us no cause for concern, we can leave the warmup parameter at its default value of 1,000.


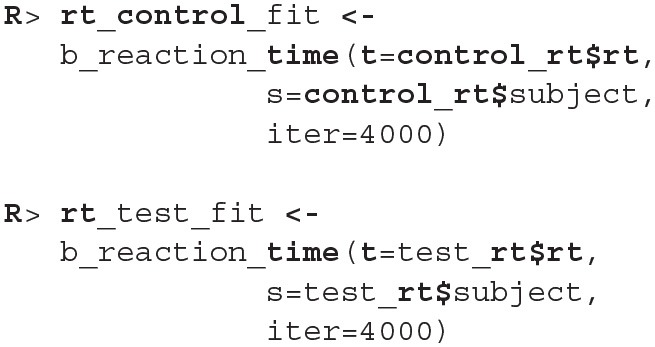


Because we did not explicitly define priors, default flat (improper) priors were used. In some cases, flat priors are a statement that we have no prior knowledge about the experiment results (in some sense). In general, even flat priors can express a preference for a certain region of parameter space. In practice, we will almost always have some prior information and we should incorporate it into the modeling process.

**Figure 7:**
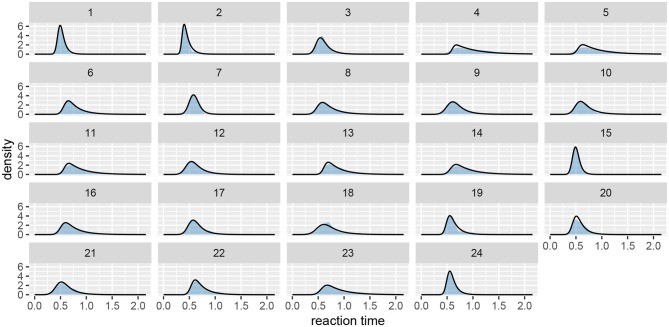
The fit plot for the rt_control_fit. The data are visualized as a blue region while the fit is visualized with a black line. In this case the model fits the underlying data well, similar conclusions can be reached for the test group (rt_test_fit).

Next, we should check whether the model fits the data well by using the plot function (see Figure 7). If we set the subjects parameter to FALSE, we will get a less detailed group level fit.


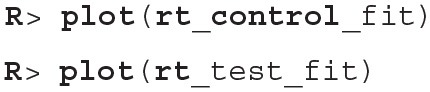


Since the model fits the data well we can move on with our analysis and use the compare_means function to compare reaction times between healthy (control) and unhealthy (test) subjects. In the example below we use a ROPE interval of 0.01 s, meaning that differences smaller that 0.01 of a second are treated as equal. The compare_means function provides us with a friendly output of the comparison and the results in the form of a data.frame.


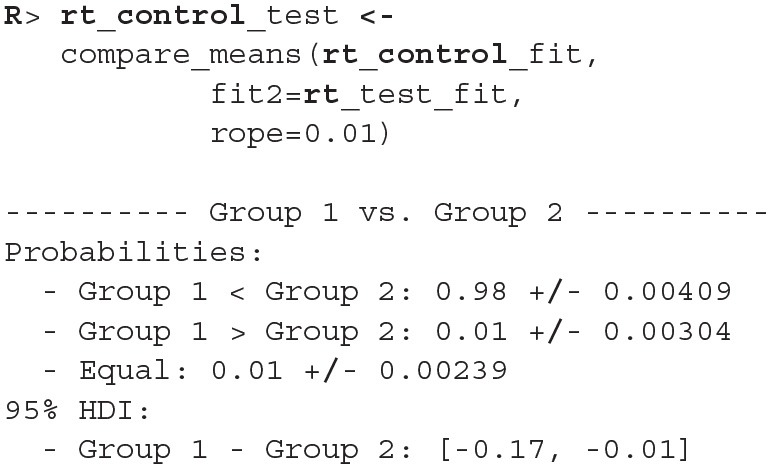


The compare_means function outputs probabilities that one group has shorter reaction times than the other, the probability that both groups are equal (if ROPE interval is provided) and the 95% HDI [highest density interval, Kruschke (2014)] for the difference between groups. Based on the output (Group 1 < Group 2) we can confidently claim (98% ± 0.4%) that the healthy group's (rt_control_fit, Group 1) expected reaction times are lower than those from the unhealthy group (rt_test_fit, Group 2).

**Figure 8:**
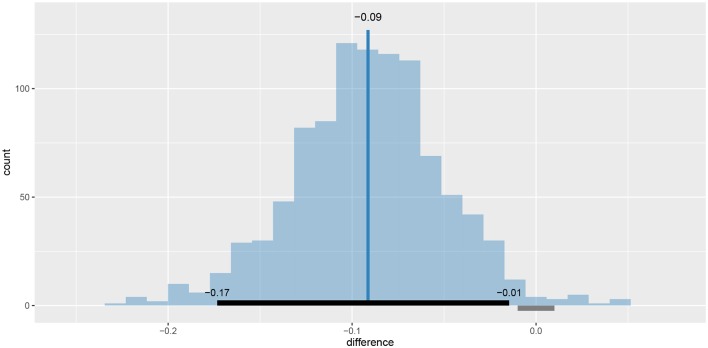
The visualization of the difference in mean reaction times between rt_control_fit and rt_test_fit. The histogram visualizes the distribution of the difference, vertical blue line denotes the mean, the black band at the bottom marks the 95% HDI interval and the gray band marks the ROPE interval. Since the entire 95% HDI of difference is negative and lies outside of the ROPE interval, we can confidently conclude that healthy subjects are faster on average.

**Figure 9:**
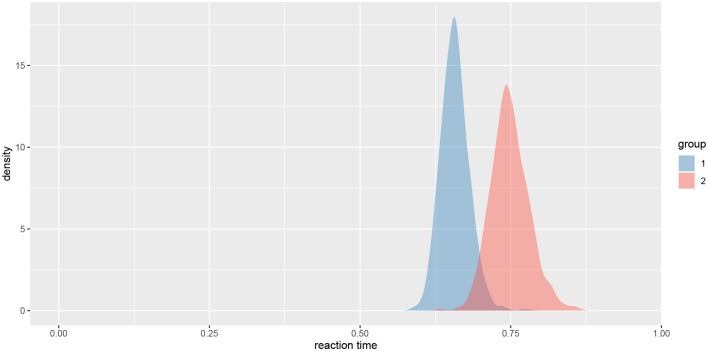
The visualization of means for rt_control_fit and rt_test_fit. Group 1 visualizes means for the healthy subjects and group 2 for the unhealthy subjects.

We can also visualize this difference with the plot_means_difference function (Figure 8), plot_means provides an alternative and visualizes the parameters that define the means of each model (Figure 9).


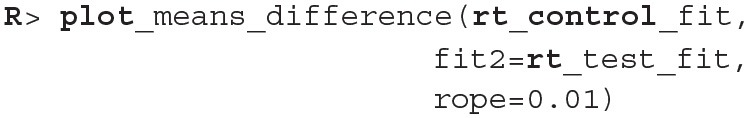



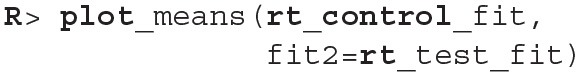


To summarize, based on our analysis we can confidently claim that healthy subjects have a lower mean reaction time when solving the flanker task than unhealthy subjects. Next, we analyse if the same applies to success rates.

The information about success of subject's is stored as correct/incorrect. However, the Bayesian success rate model requires binary (0-1) inputs so we first have to transform the data. Also, just like in the reaction time example, we have to correct the indexes of control group subjects.


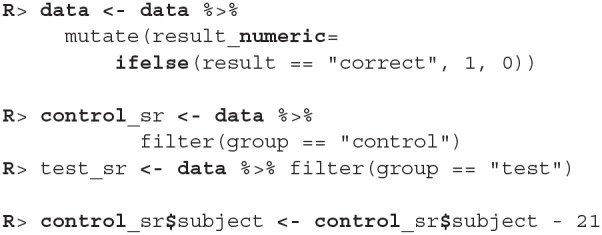


Since the only prior information we have about the success rate of participants is that it is between 0 and 1, we used a beta distribution to put a uniform prior on the [0, 1] interval (we put a Beta(1, 1) prior on the *p* parameter). We fit the model by running the b_success_rate function with appropriate input data.


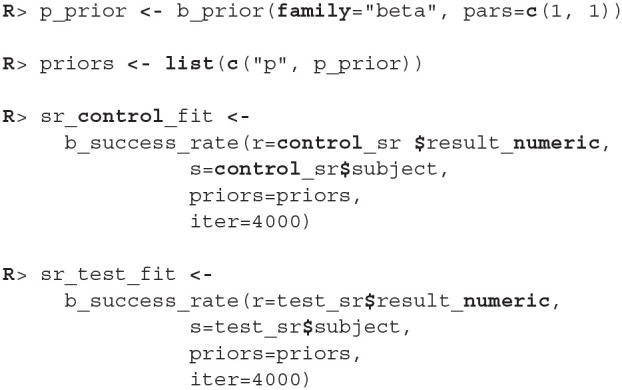


The process for inspecting Bayesian fits (through plot_trace and print functions) is the same and since the results are similar as above we omitted them here. When visually inspecting the quality of the fit (the plot function) we can set the subjects parameter to FALSE, which visualizes the fit on the group level. This offers a quicker, but less detailed method of inspection.


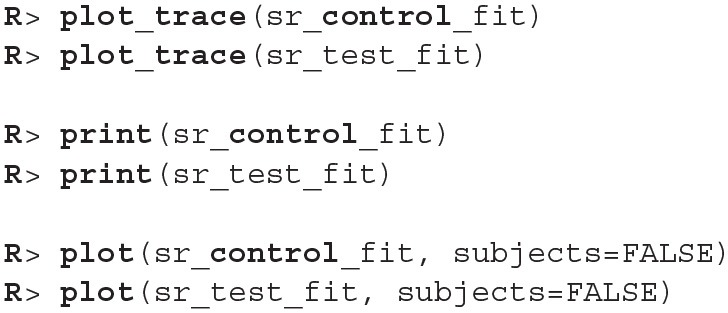


Since diagnostic functions show no cause for concern and the fits look good we can proceed with the actual comparison between the two fitted models. We will again estimate the difference between two groups with compare_means.


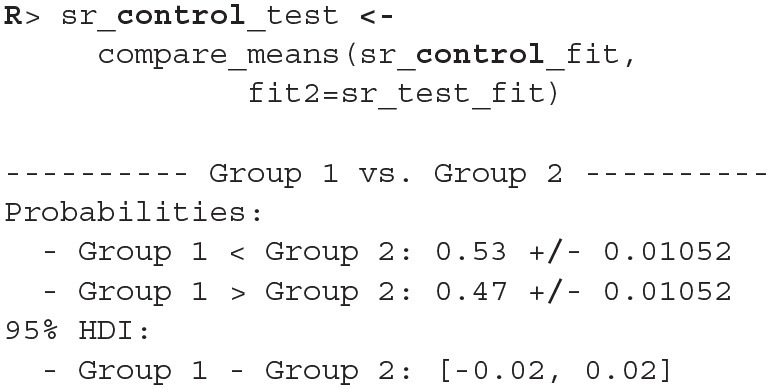


**Figure 10:**
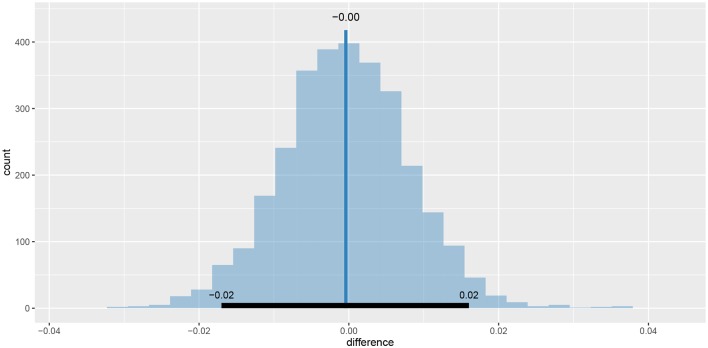
The visualization of the difference between sr_control_fit and sr_test_fit. The histogram visualizes the distribution of the difference, vertical blue line denotes the mean difference and the black band at the bottom marks the 95% HDI interval. Since the 95% HDI of difference includes 0 we cannot claim inequality. If we used a ROPE interval and the whole ROPE interval lied in the 95% HDI interval we could claim equality.

As we can see the success rate between the two groups is not that different. Since the probability that healthy group is more successful is only 53% (± 1%) and the 95% HDI of the difference ([−0.02, 0.02]) includes the 0 we cannot claim inequality (Kruschke, 2014). We can visualize this result by using the plot_means_difference function (Figure 10).


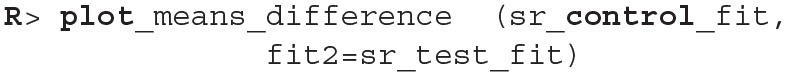


### 3.2. Adaptation Level

In the adaptation level experiment participants had to assess weights of the objects placed in their hands by using a verbal scale: very very light, very light, light, medium light, medium, medium heavy, heavy, very heavy, and very very heavy. The task was to assess the weight of an object that was placed on the palm of their hand. To standardize the procedure the participants had to place the elbow on the desk, extend the palm and assess the weight of the object after it was placed on their palm by slight up and down movements of their arm. During the experiment participants were blinded by using non-transparent fabric. In total there were 15 objects of the same shape and size but different mass (photo film canisters filled with metallic balls). Objects were grouped into three sets:

- the light set: 45, 55, 65, 75, 85 g (weights 1–5),
- the medium set: 95, 105, 115, 125, 135 g (weights 6–10),
- the heavy set: 145, 155, 165, 175, 185 g (weights 11–15).

The experimenter sequentially placed weights in the palm of the participant and recorded the trial index, the weight of the object and participant's response. The participants were divided into two groups, in group 1 the participants first assessed the weights of the light set in ten rounds within which the five weights in the set were weighted in a random order. After completing the 10 rounds with the light set, the experimenter switched to the medium set. The participant then weighted the medium set across another 10 rounds of weighting the five weights in the medium set in a random order. In group 2 the overall procedure was the same, the only difference being that they started with the 10 rounds of the heavy set and then performed another 10 rounds of weighting on the medium set. Importantly, the weights within each set were given in random order and the experimenter switched between sets seamlessly without any break or other indication to the participant.

We will use the bayes4psy package to show that the two groups provide different assessment of the weights in the second part of the experiment even though both groups are responding to weights from the same (medium) set. We will use Bayesian analysis to test the hypothesis that in the second part of the experiment the difference is very pronounced at first but then fades away with subsequent assessments of weights from the medium set. This is congruent with the hypothesis that each group formed a different adaptation level during the initial phase of the task, the formed adaptation level then determined the perceptual experience of the same set of weights at the beginning of the second part of the task.

We will conduct the analysis by using the hierarchical linear model. First we have to construct fits for the second part of the experiment for each group independently. The code below loads and prepares the data, just like in the previous example, subject indexes have to be mapped to a [1, n] interval. We will use the ggplot2 package to fine-tune graph axes and properly annotate graphs returned by the bayes4psy package.


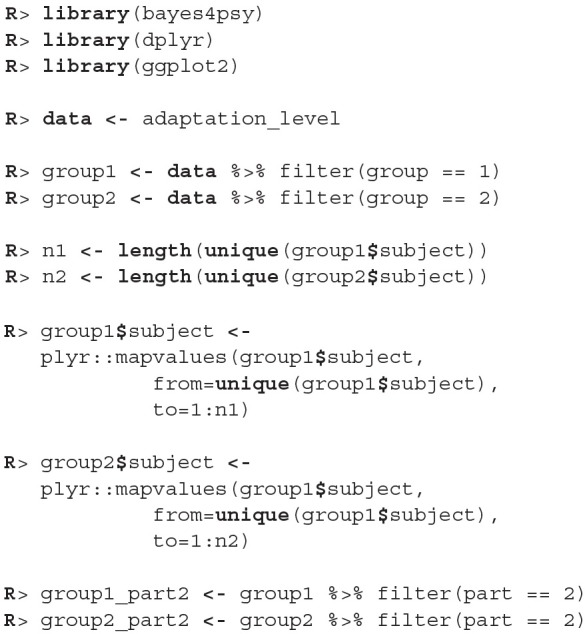


Once the data is prepared we can start fitting the Bayesian models, the input data comes in the form of three vectors, *x* stores indexes of the measurements, *y* the subject's responses and *s* indexes of the subjects. The warmup and iter parameters are set in order to achieve an effective sample size of 10,000.


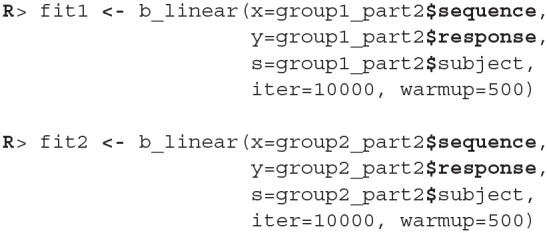


The fitting process is always followed by the quality analysis.


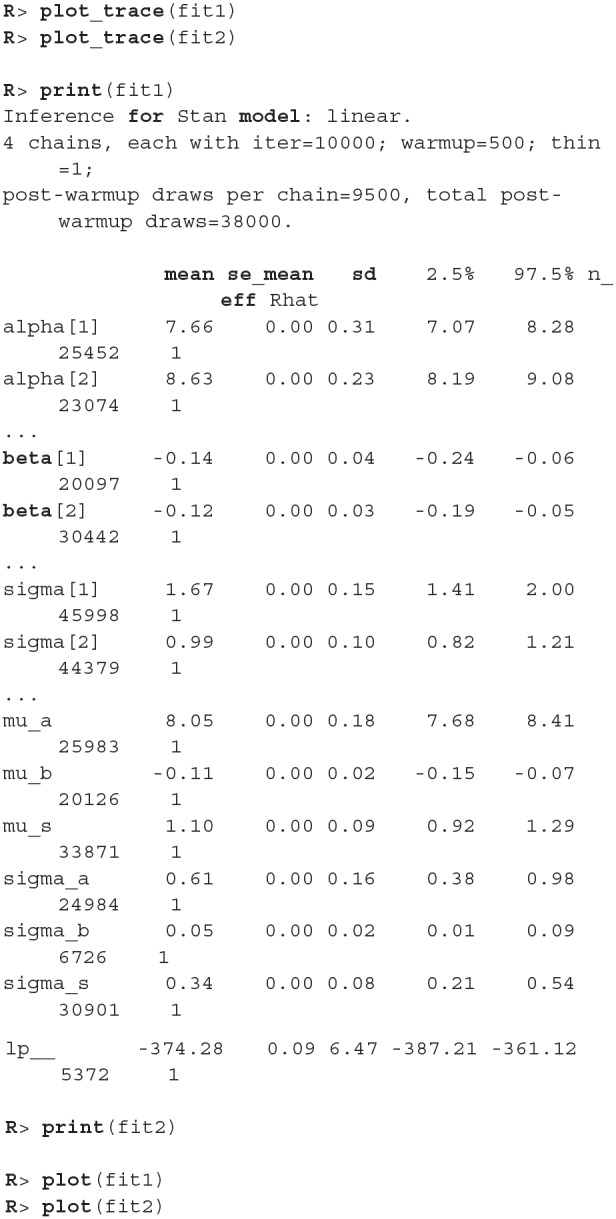


The trace plot showed no MCMC related issues (for an example of trace plot see Figure 6), effective sample sizes of parameters relevant for our analysis (μ_*a*_, μ_*b*_, and μ_*s*_) are large enough. Since the visual inspection of the fit also looks good we can continue with our analysis. To get a quick description of fits we can take a look at the summary statistics of the model's parameters.


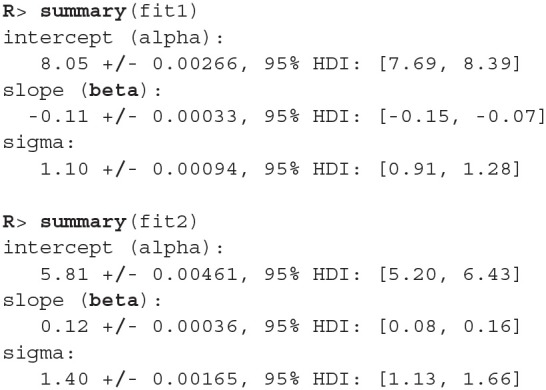


**Figure 11:**
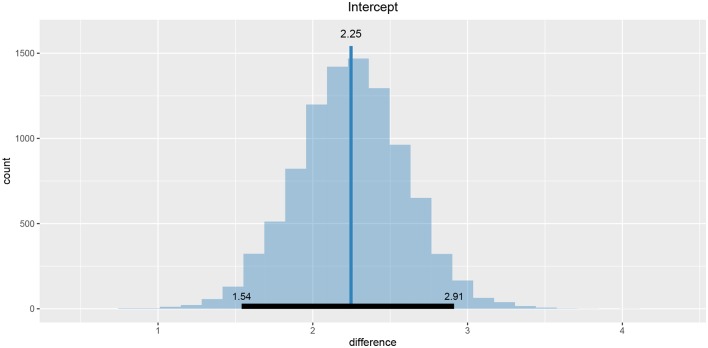
The difference between the intercept of the two fits. Since the entire 95% HDI is positive we are confident that the subject's that weighted lighter object in the first part of the experiment (fit1) find medium objects heavier than subjects that initially weighted heavier objects (fit2).

Values of intercept (95% HDI intercept equals [7.69, 8.39] for the first group and [5.20, 6.43] for the second group) suggest that our initial hypothesis about adaptation level is true. Subject's that weighted lighter object in the first part of the experiment (fit1) find medium objects at the beginning of experiment's second part heavier than subjects that weighted heavier objects in the first part (fit2). We can confirm this assumption by using functions that perform a more detailed analysis (e.g., compare_means and plot_means_difference, see the output below and Figure 11).


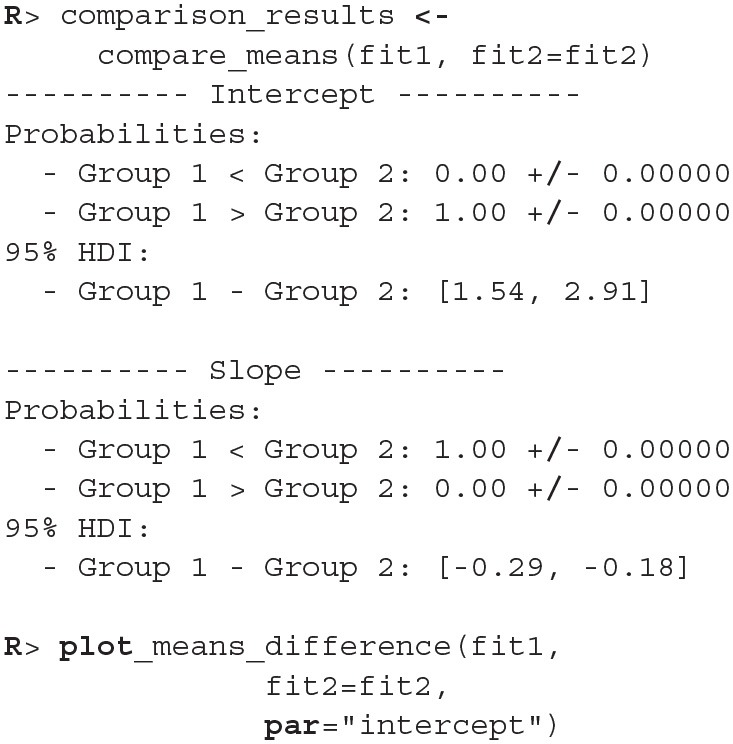


**Figure 12:**
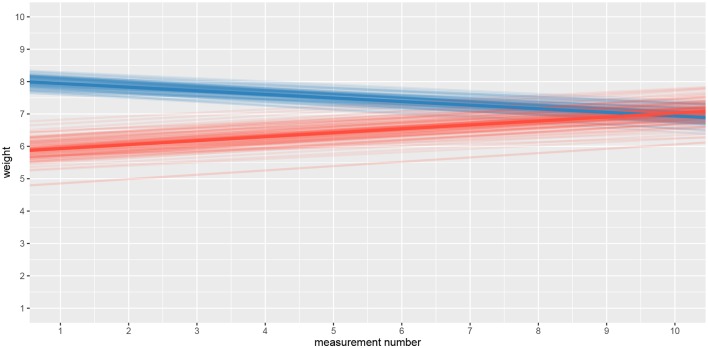
Comparison of distributions underlying fit1 and fit2. The hypothesis that each group formed a different adaptation level during the initial phase of the task seems to be true. The group that switches from heavy to medium weights assesses weights as lighter than they really are, while for the group that switches from light to medium the weights appear heavier. These adaptation levels fade with time and assessments converge to similar estimates of weights.

The fact that we are confident in the claims that the slope for the first group is negative (95% HDI for the first group's slope equals [−0.15, −0.07] and lies entirely below 0) and positive for the second group (95% HDI for the second group's slope equals [0.08, 0.16] and lies entirely above 0) suggests that the adaptation level phenomenon fades away with time. We can visualize this by plotting means and distributions underlying both fits. The plotting functions in the bayes4psy package return regular ggplot2 plot objects, so we can use the same techniques to annotate or change the look and feel of graphs as we would with the usual ggplot2 visualizations (see the code below and Figure 12).


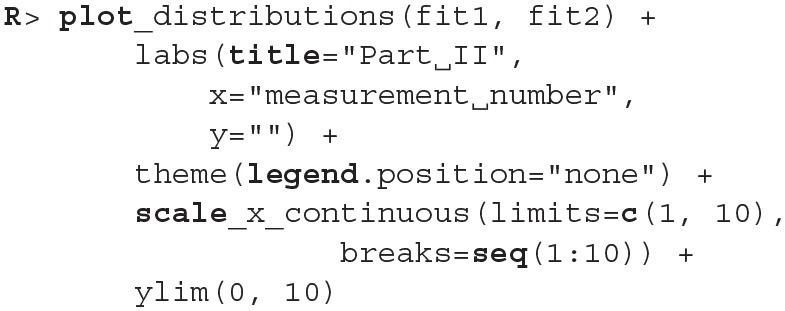


### 3.3. The Stroop Color-Word Test

The Stroop test (Stroop, 1935) showed that when the stimuli are incongruent—the name of a color is printed in different ink than the one denoted by its name (for example, red)—naming the color takes longer and is more error-prone than naming the color of a rectangle or a set of characters that does not form a word (for example, XXXXX).

In our version of the Stroop test participants were faced with four types of conditions:

- Reading neutral—the name of the color was printed in black ink, the participant had to read the color's name.
- Naming neutral—string XXXXX was written in colored ink (red, green or blue), the participant had to name the ink color.
- Reading incongruent—name of the color was printed in incongruent ink, the participant had to read the written name of the color.
- Naming incongruent—name of the color was printed in incongruent ink, the participant had to name the ink color.

In each of the listed conditions the participants had to name or read 100 stimuli presented on an A4 sheet of paper organized in 5 columns of 20 stimuli as quickly as possible. The specific order of the stimuli was pseudo-random and balanced across the sheet. We recorded the time to complete each sheet.

In our example analysis, we are primarily interested in expected task completion times. Since our data is composed from average times needed to complete the task we can use the Bayesian *t*-test. The nature of the Stroop test requires the use of *t*-test for dependent samples. This example first shows how to execute the Bayesian *t*-test for dependent samples and in the second part, for illustrative purposes only, also how to execute the Bayesian *t*-test for independent samples. The example for independent samples also shows how to use bayes4psy to compare multiple groups simultaneously.

To execute the Bayesian *t*-test for dependent samples we first have to calculate the difference between the samples and then perform Bayesian modeling on those differences. The example below compares reading times between neutral and incongruent conditions.


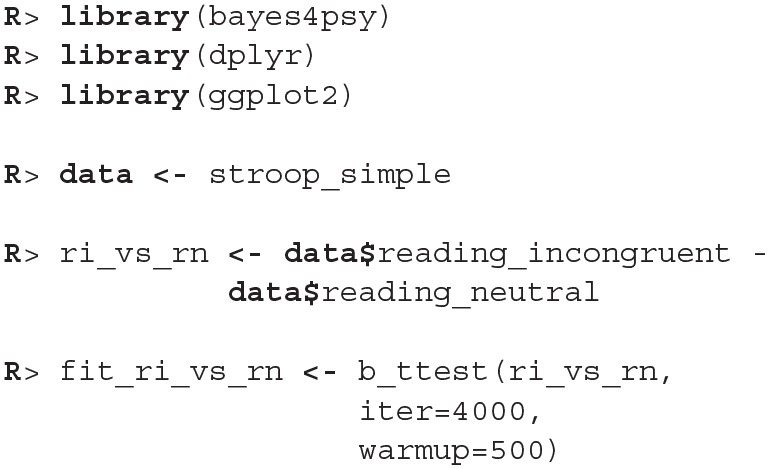


Once we fit the Bayesian *t*-test model to the differences between the reading neutral and reading incongruent conditions, we can compare whether the means differ from 0.


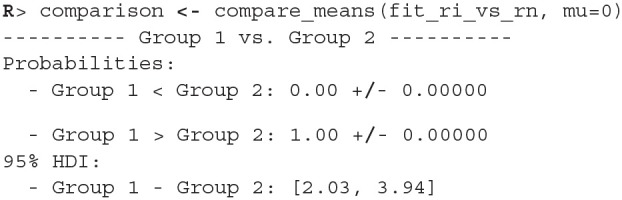


Since the 95% HDI of means ([2.03, 3.94]) lies above 0 we can confidently claim that subject's read neutral stimuli faster than incongruent stimuli. In a similar fashion we can also execute a comparison between other conditions.

The examples that follow are for illustrative purposes only, they analyse the Stroop data under the wrongful assumption that the samples are independent. These examples are in the manuscript mainly to explain how we can use bayes4psy to compare multiple groups simultaneously. The examples also include priors, we based them on our previous experience with similar tasks—participants finish the task in ~1 min and the typical standard deviation for a participant is <2 min.


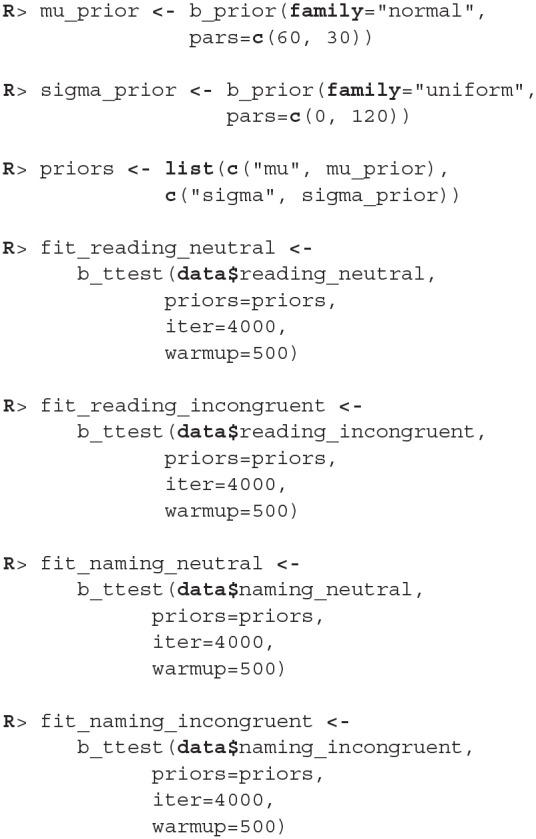


There were no causes for concern in the MCMC diagnostics and model fits, so we omit them for brevity. In practice, we should of course always perform these steps. We proceed by cross-comparing several fits with a single line of code.


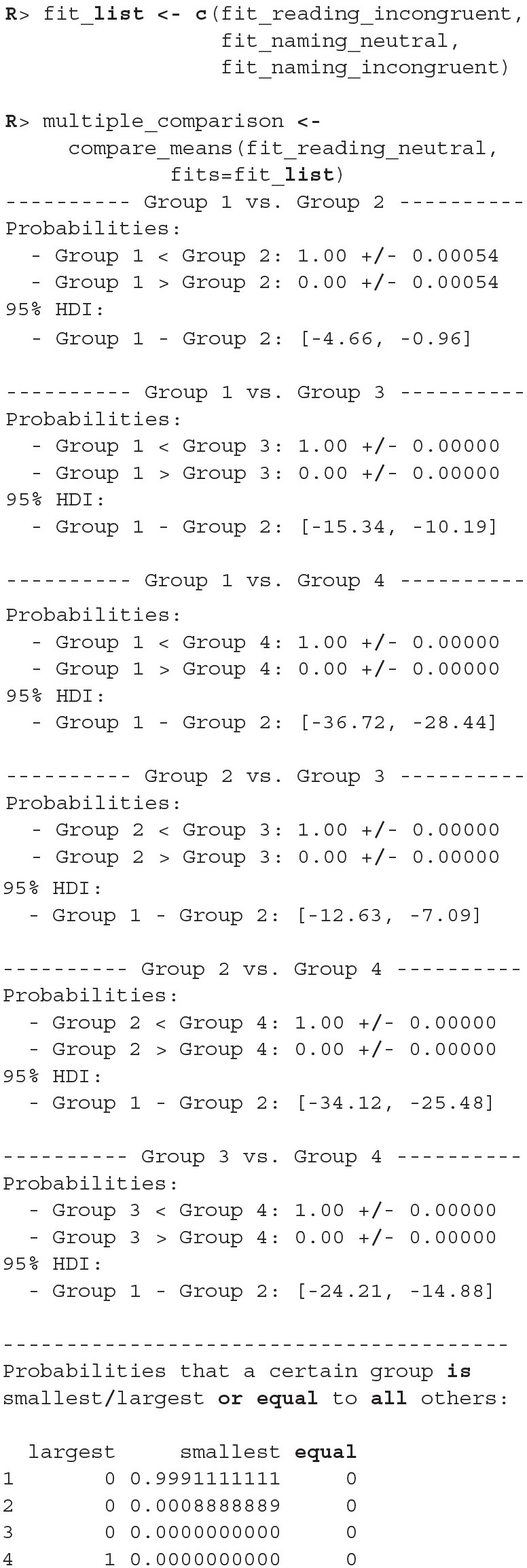


**Figure 13:**
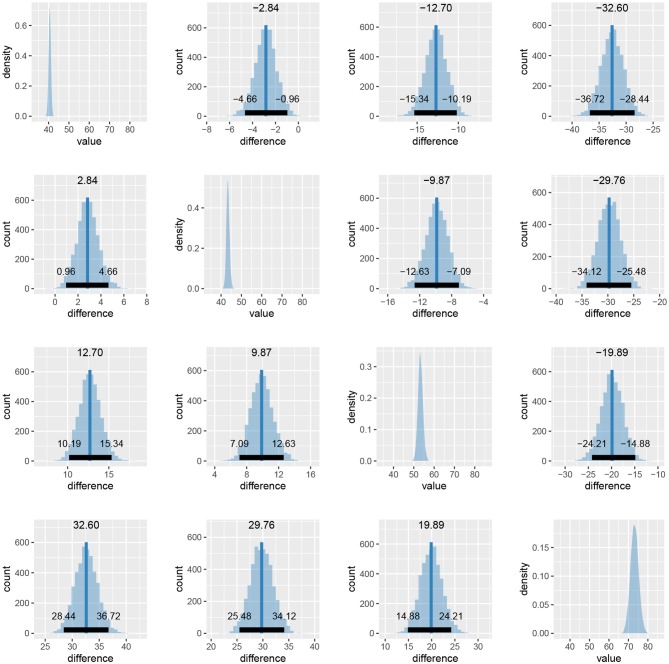
Differences in the mean task completion times for the four conditions. Row and column 1 represent the reading neutral task, row and column 2 the reading incongruent task, row and column 3 the naming neutral task and row and column 4 the naming incongruent task. Since 95% HDI intervals (black bands at the bottom of graphs) in all cases exclude 0 we are confident that the task completion times between conditions are different.

When we compare more than two fits, we also get an estimate of the probabilities that a group has the largest or the smallest expected value. Based on the above output, the participants are best at the reading neutral task (Group 1), followed by the reading incongruent task (Group 2) and the naming neutral task (Group 3). They are the worst at the naming incongruent task (Group 4). We are very confident that this ordering is correct (the probabilities distinguishing the groups are extremely high), so we can conclude that both naming and incongruency of stimuli increase the response times of subjects, with naming having a bigger effect. We can also visualize this in various ways, either as distributions of mean times needed to solve the given tasks or as a difference between these means (Figure 13).


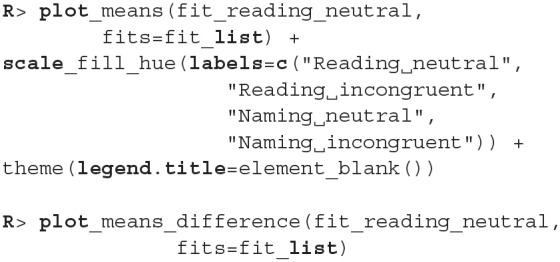


### 3.4. Afterimages

In the afterimages task participants were asked to fix their gaze on a fixation point in the middle of the computer screen. Stimulus—a colored rectangle—was then shown above the fixation point. After 20 s the rectangle disappeared and a color palette was shown on the right-hand side of the screen. Participants were asked to keep their gaze on the fixation point while using the mouse to select the color that best matched the color of the afterimage that appeared above the fixation point. To help select the correct color, a rectangle of the same size as the adapting stimuli was shown below the fixation point in the color currently under the mouse cursor. Participants confirmed their selection by pressing a mouse button when they were satisfied that color of the rectangle below the fixation point matched the color of the afterimage experienced above the fixation point. For each trial the color of the stimulus rectangle, the subject's response in RGB and the subject's response time were recorded. The goal of this study was to determine which of the two color coding mechanisms (trichromatic or opponent-process) better explains the perceived color of the afterimages. We used six differently colored rectangles: red, green, blue, cyan, magenta, yellow.

We start our analysis by loading the experiment and stimuli data. The experiment data include subject index, reaction time, response in RGB format, stimuli name (e.g., blue) and stimuli values in RGB and HSV. The stimuli data include the information about stimuli (stimuli names and their RGB/HSV values).


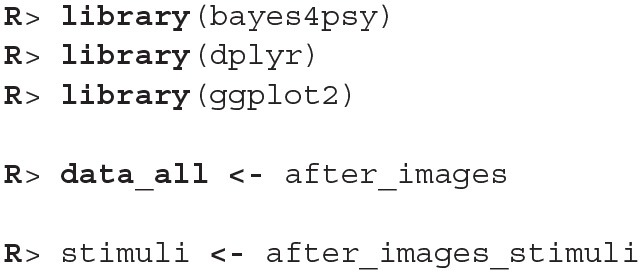


**Figure 14:**
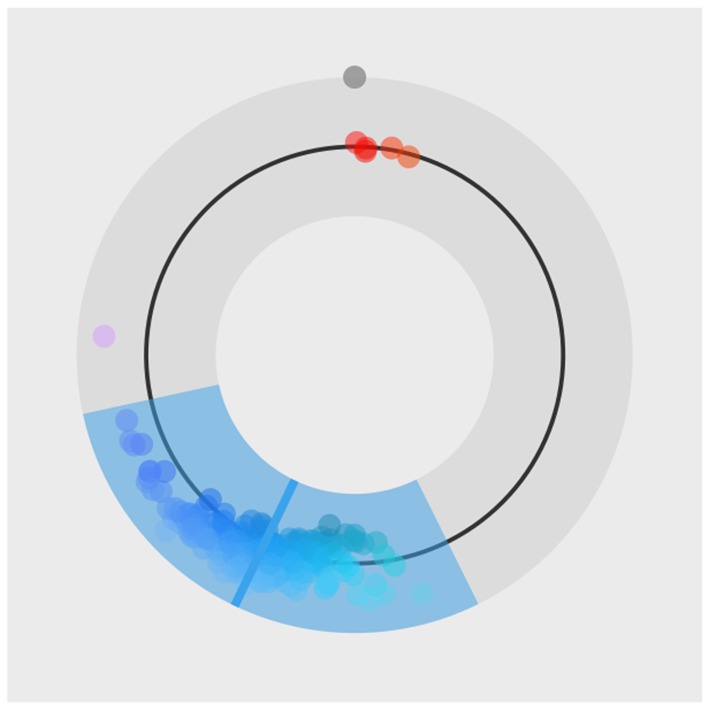
The special plot_hsv function developed for the color model. Input data points are visualized with circles, mean of the fit is visualized with a solid line and the 95% HDI of the underlying distribution is visualized as a colored band.

Once we load required libraries and data we can start fitting Bayesian color models. Below is a detailed example of fitting the Bayesian color model for the red color stimuli. For a visual inspection of the fit (see Figure 14).


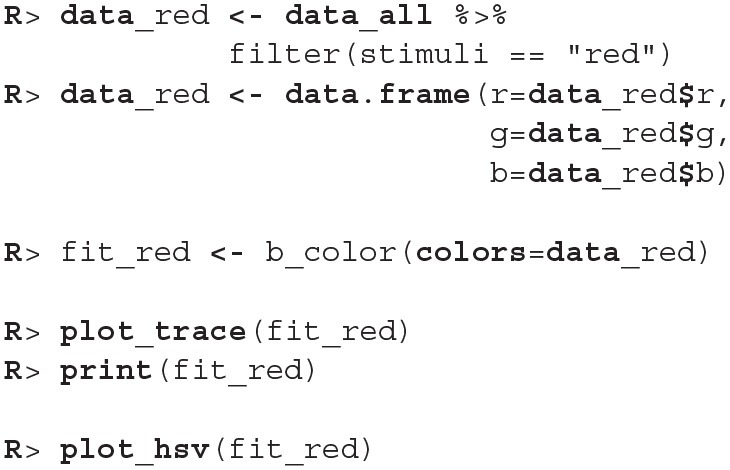


We repeat the same process five more times for the remaining five colors of stimuli. We start the analysis by loading data about the colors predicted by the trichromatic and the opponent-process theory.


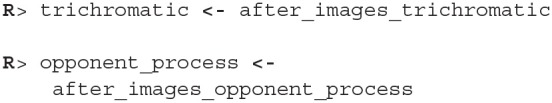


We can then use the plot_distributions_hsv function of the Bayesian color model to produce a visualization of the accuracy of both color coding mechanism predictions for each stimuli independently. Each graph visualizes the inferred distribution, displayed stimuli, and responses predicted by the trichromatic and opponent-process coding. This additional information can be added to the visualization via annotation points and lines. Below is an example for the red stimulus, visualizations for other five stimuli are practically the same.


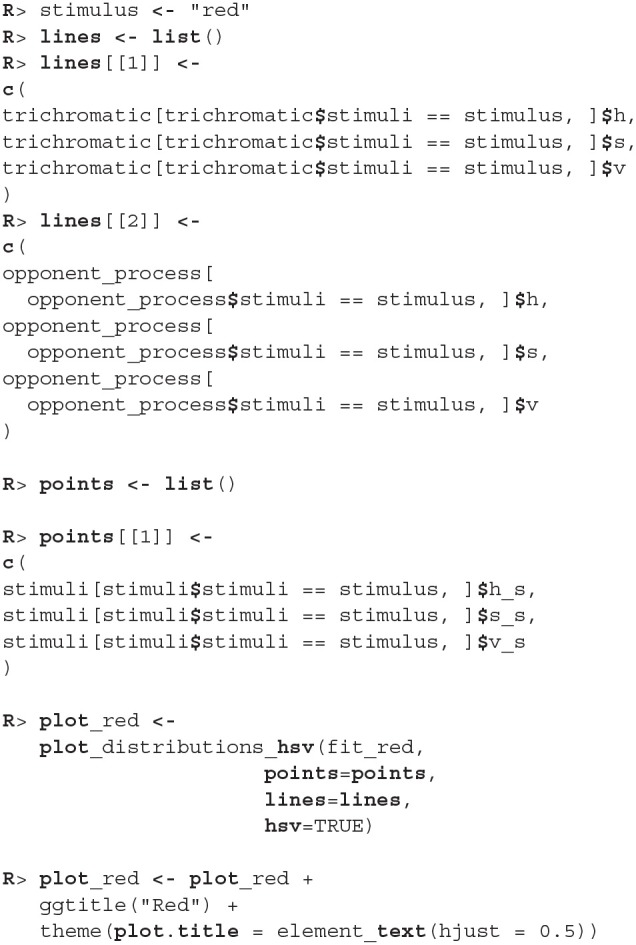


**Figure 15:**
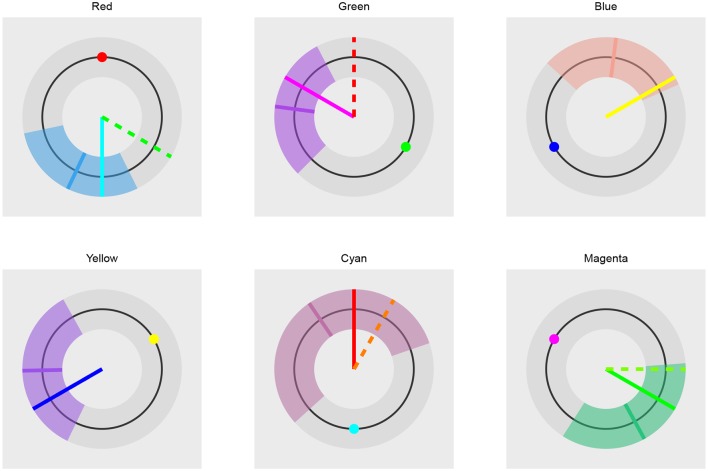
The comparison of trichromatic and opponent-process color coding prediction. The long solid line visualizes the trichromatic color coding prediction. The dashed line visualizes the opponent-process color coding prediction. Short solid line represents the mean hue of the fit. The colored band the 95% HDI of the distribution underlying the fit. The small colored circle visualizes the color of the presented stimuli. In the case of blue and yellow stimuli the dashed line is not visible because both color codings predict the same outcome. The prediction based on the trichromatic color coding seems more accurate as its prediction is always inside the 95% of the most probable subject's responses and is always closer to the mean predicted hue than the opponent-process prediction. The opponent-process prediction is outside of the 95% of the most probable subject's responses in cases of red and green stimuli.

We can use the cowplot (Wilke, 2019) package to combine the plots into a single figure (see Figure 15).


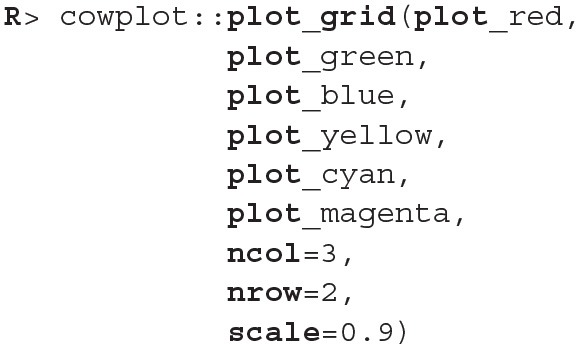


## 4. Discussion

The bayes4psy package helps psychology students and researchers with little or no experience in Bayesian statistics or probabilistic programming to do modern Bayesian analysis in R. The package includes several Bayesian models that cover a wide range of tasks that arise in psychological experiments. We can perform a Bayesian *t*-test or Bayesian bootstrap, analyse reaction times, success rates, colors, or sequential tasks. The package covers all parts of Bayesian data analysis, from fitting and diagnosing fitted models to visualizations and comparisons.

We plan to continuously upgrade the package with new tools and Bayesian statistics even closer to non-technical researchers. For example, we will implement probability distribution elicitation tools, which will ease the extraction of prior knowledge from domain experts and the prior construction process (Morris et al., 2014). Over the last couple of years neuroimaging techniques (e.g., fMRI and EEG) have become very popular for tracking brain activity during psychological experiments. The implementation of Bayesian models for analysing such data is also one of our future goals.

## Data Availability Statement

The results in this paper were obtained using R 3.5.3. Core R and all packages used are available from the Comprehensive R Archive Network (CRAN) at https://CRAN.R-project.org/.

The source code of bayes4psy can be found at https://github.com/bstatcomp/bayes4psy and the illustrative examples from section 3 are included in the package as vignettes. The bayes4psy package is also published on the CRAN repository (https://cran.r-project.org/package=bayes4psy).

## Author Contributions

JD, GR, and EŠ designed the study. GR determined which models should be implemented and gathered and prepared example data for these models. JD with supervision and guidance from EŠ developed the package and Bayesian models. JD prepared the illustrative examples. All the authors wrote the paper.

## Conflict of Interest

The authors declare that the research was conducted in the absence of any commercial or financial relationships that could be construed as a potential conflict of interest.
